# Iodine deficiency in pregnancy along a concentration gradient is associated with increased severity of preeclampsia in rural Eastern Cape, South Africa

**DOI:** 10.1186/s12884-021-04356-6

**Published:** 2022-02-04

**Authors:** Charles Bitamazire Businge, Benjamin Longo-Mbenza, Andre Pascal Kengne

**Affiliations:** 1 Department of Obstetrics and Gynaecology, Faculty of Health Sciences, Walter Sisulu University, Private Bag x1 WSU, Mthatha, 5117 South Africa; 2 Department of Medicine, Faculty of Health Sciences, University of Cape Town, Cape Town, South Africa; 3 Faculty of Medicine, University of Kinshasa, Kinshasa, Democratic Republic of Congo; 4 LOMO University of Research, Kinshasa, Democratic Republic of Congo; 5 Non-Communicable Disease Research Unit, South African Medical Research Council, Cape Town, South Africa

## Abstract

**Background:**

Preeclampsia is a leading cause of maternal mortality and morbidity in South Africa. Iodine deficiency in pregnancy, which is amenable to correction through iodine supplementation, has been reported to increase the risk of preeclampsia. However, the association of iodine nutrition status with preeclampsia in South Africa has not been studied.

**Methods:**

We enrolled 51 randomly selected normotensive pregnant controls at term together with 51 consecutively selected cases of preeclampsia and 51 cases of severe preeclampsia/eclampsia, all in the third trimester, from Mthatha Regional and Nelson Mandela Academic Hospital in the Eastern Cape Province. Urinary iodine concentration (UIC), serum thyroid-stimulating hormone (TSH), triiodothyronine (FT3), thyroxine (FT4) and thyroglobulin (Tg) levels were compared between cases and controls.

**Results:**

The respective chronological and gestational ages at enrolment for normotensive, preeclampsia and severe preeclampsia/eclampsia participants were: age 23, 24 and 19 years (*p* = 0.001), and gestational age 38, 34, and 35 weeks (*p* < 0.001). The median gravidity was 1 for all three groups. The median UIC, FT4, FT3 revealed a decreasing and Tg a rising trend with the severity of preeclampsia (*p* < 0.05). TSH had a non-significant rising trend (*p* > 0.05). The respective median values for normotensive, preeclampsia and severe preeclampsia/eclampsia participants were UIC 217.1, 127.7, and 98.8 μg/L; FT4 14.2, 13.7, and 12. pmol/L; FT3 4.8, 4.4, and 4.0 pmol//L; Tg 19.4, 21.4, and 32. Nine microgram per liter; TSH 2.3, 2.3, and 2.5 mIU/L. UIC < 100 μg/L, Tg > 16 μg/L and FT4 < 11.3 pmol/L were independent predictors of preeclampsia/eclampsia syndrome.

**Conclusion:**

Women with severe preeclampsia/eclampsia had significantly low UIC and high Tg, suggesting protracted inadequate iodine intake. Inadequate iodine intake during pregnancy severe enough to cause elevated Tg and FT4 deficiency was associated with an increased risk of severe preeclampsia/eclampsia.

## Background

Preeclampsia is a leading cause of maternal and perinatal morbidity and mortality around the world, including in South Africa [1, 2]. The foetal complications of preeclampsia arise from placenta ischaemia. These include intrauterine growth restriction, intrauterine foetal death, premature birth with attendant perinatal morbidity and mortality, increased risk of metabolic syndrome in adult life [2, 3]. The maternal complications of preeclampsia arise from endothelial dysfunction, leading to multisystem organ dysfunction. This dysfunction includes thrombocytopenia, haemolysis, hepatocellular injury, pulmonary oedema, acute kidney failure, cerebral oedema, cerebral haemorrhage, eclamptic fits, future cardiovascular disease and maternal death [2, 3].

The exact cause of preeclampsia is not known with certainty [4]. However, several risk factors have now been identified [2]. Thyroid dysfunction in pregnancy has also been identified as one of the risk factors for preeclampsia [5]. Particularly, overt and subclinical hypothyroidism are increasingly being recognised as risk factors for preeclampsia [5–7]. Hypothyroidism in pregnancy also predisposes to increased risk of maternal-anaemia, caesarean delivery, post-partum haemorrhage, miscarriage, low birth weight, low Apgar score and neonatal intensive care admission [8, 9]. Chronic iodine deficiency is associated with an increased risk of hypothyroidism [10]. In addition, an association has been reported between iodine deficiency and preeclampsia potentially mediated through reduced antioxidant capacity and or prolonged stimulation of the endothelium by elevated levels of thyroid-stimulating hormone [11, 12]. Therefore, the increase in renal filtration of iodine secondary to the physiological increase in renal blood flow, and the progressive transfer of iodine to the foetus [13] may predispose pregnant women with low daily iodine intake to iodine deficiency disorders and incident preeclampsia.

The incidence of preeclampsia in the general population of South Africa, and specifically in the Eastern Cape, is not fully known. In one study among South African primigravida women, Moodley et al. [14] reported an incidence of preeclampsia-eclampsia syndrome of about 5.8% with a resultant perinatal mortality rate of 5.9% compared to 2.2% in the general population. In another study in the KwaZulu-Natal Province, Panday et al. [15] reported an incidence of hypertensive disease in pregnancy of 12.5% at the community level and 14.5% at the referral tertiary hospital. In South Africa, hypertensive disease in pregnancy accounts for about 22% of all preventable maternal deaths [16].

Before implementing universal salt iodization, South Africa had endemic iodine deficiency, which was the underlying cause of goitre in the population [17, 18]. Despite the initiation of universal salt iodization around 1995, about 30% of the population does not have regular access to adequately iodised salt, especially in rural communities [17, 18]. Since pregnancy is associated with increased physiological iodine loss, undiagnosed iodine deficiency in pregnancy is likely prevalent in South Africa. Although the association between iodine deficiency and preeclampsia has been reported in some studies [11, 12], such an association has not yet been investigated in South Africa. If iodine deficiency in pregnancy, which can be mitigated with iodine supplementation, is found to be a risk factor of preeclampsia in this population, efforts to improve the iodine nutrition state in reproductive years may help reduce the burden of preeclampsia.

Therefore, we carried out this study in order to 1) find out the iodine nutrition status of normotensive pregnant women and their counterparts with hypertensive disease in pregnancy; 2) find out whether iodine deficiency is associated with increased risk and severity of preeclampsia; and 3) investigate whether women with preeclampsia/eclampsia had a significant elevation of thyroid-stimulating hormone (TSH), which is one of the pathological mechanisms of preeclampsia; as well as determine serum nitric oxide levels, a marker of antioxidant capacity and endothelial dysfunction.

## Materials and methods

### Study setting and population

**Fig. 1:**
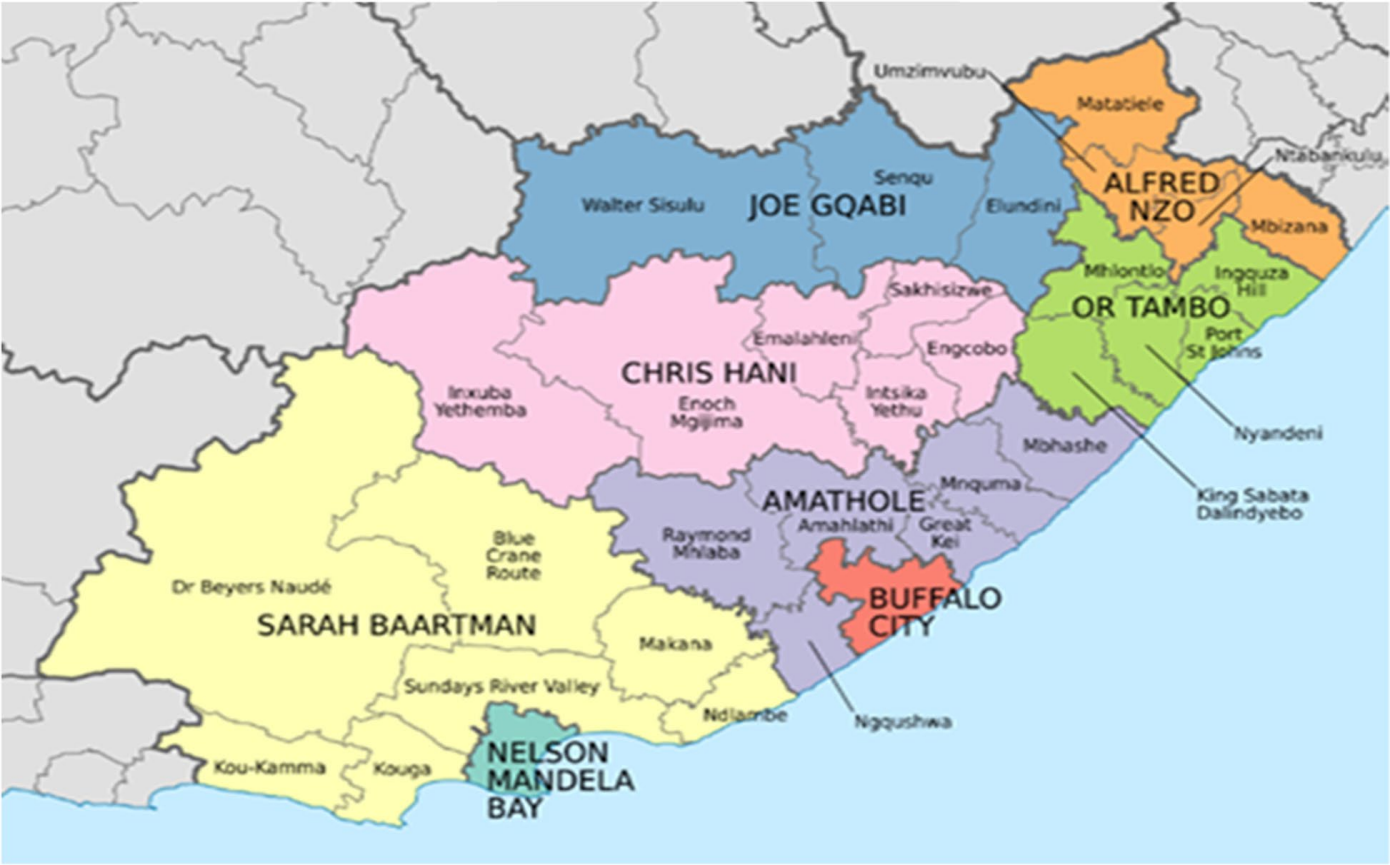
Map of the Eastern Cape Province, South Africa, showing OR Tambo and other district municipalities

We conducted this study at two hospitals in OR Tambo District Municipality (ORTDM), Eastern Cape, South Africa. Both hospitals provide care to patients referred from district hospitals and Community Health Centres in the north-eastern part of the Eastern Cape Province South Africa (Fig. 1), a region with a high incidence of preeclampsia/eclampsia. Mthatha Regional Hospital (MRH) is a 500-bed referral hospital for the district hospitals and health centres in the King Sabata Dalindyebo Health sub-district (KSD) within ORTDM. It has a maternity unit of 80 beds that mainly conducts normal deliveries and emergency and elective caesarean sections for low-risk antenatal mothers, and refers high-risk obstetrics patients to Nelson Mandela Academic Hospital (NMAH). NMAH is an 800-bed teaching/tertiary hospital with 100 maternity beds also in KSD. About 4500 births are conducted at the NMAH labour ward, out of which 40% are mothers whose pregnancies are complicated with hypertensive diseases in pregnancy.

This north-eastern part of the Eastern Cape Province (the former Transkei region) is largely composed of rural and peri-urban human settlements with a population of about 1.8 million people. Seventy-five per cent of the population is not gainfully employed and therefore considered being below the poverty line. This is an area formerly with endemic goitre before implementing salt iodisation in 1995 [17]. Although iodization of salt was implemented in South Africa since 1995, the consumption of iodised salt for some segments of the population including women of reproductive age could be inadequate considering that some rural communities are more inclined to use cheaper non-iodised salt [18]. Second, the peri-urban communities are more likely to depend on foodstuffs from the food chain stores considering the level of globalisation [19]. These have a high probability of having been processed without using iodised salt, and could also have a high content of perchlorate, which is a common ingredient of fertilisers and an inhibitor of thyroid iodine uptake, exposing the population to the risk of iodine deficiency which is more apparent during pregnancy [20].

### Study design

This was a case-control study carried out between August 2018 and March 2020. Women, referred to NMAH with preeclampsia/eclampsia and normotensive women without chronic medical diseases reporting for delivery at Mthatha Regional Hospital and Nelson Mandela Academic Hospital, who fulfilled the study criteria were eligible for enrolment.

### Sample size

We determined the sample size (N) for the study as follows [21]:

$$\mathrm N=\left[\left(1/{\mathrm q}_1+1/{\mathrm q}_2\right)\mathrm{SD}^2\left({\mathrm z}_{\mathrm\alpha+}{\mathrm z}_{\mathrm\beta}\right)^2\right]/\mathrm E^2$$

Where:

z_α_ is the standard normal deviate of α (0.05) = 1.96.

z_β_ is the standard normal deviate of β (0.20) = 0.84.

q1 = ½ (the proportion of participants in study group 1).

q2 = ½ (the proportion of participants in study group 2).

SD = the standard deviation.

E = the effect size (mean UIC of controls cases minus mean UIC of cases).

N = total number of study participants.

Ceullar-Rufino [9] found that the urinary iodine concentration (UIC) of normotensive pregnant women was 185.7 ± 77.16 μg/L, while the UIC of women with hypertensive disease in pregnancy was 142.15 ± 84.8 μg/L. The difference in the two means (E) was 42.3 μg/L. Assuming a difference in mean UIC (E) of cases with preeclampsia and normotensive pregnant controls in our study population of 45 μg/L and a standard deviation of 80 μg/L. Using this data and the formula above, N, the total number of participants in two comparison groups was 99.1. Hence, each group of cases or controls would comprise at least 50 participants.

We enrolled 51 normotensive pregnant controls, and 51 newly diagnosed participants in each group of preeclampsia without severe features, and preeclampsia with severe features/eclampsia cases. We assigned the cases the diagnosis of eclampsia, preeclampsia with or without severe features, according to the International Society for the Study of Hypertension in Pregnancy (ISSHP) guidelines [22]. Normotensive pregnant women admitted at term for elective caesarean section and counterparts in latent labour at term admitted for vaginal delivery were enrolled as controls.

### Inclusion and exclusion criteria

All consenting women who fulfilled the definition of cases or controls were eligible to take part in the study. Women with multiple pregnancy, those with a history of thyroid disease, renal disease, chronic hypertension or diabetes mellitus were excluded.

### Sampling

We enrolled the participants with preeclampsia and eclampsia soon after diagnosis and referral to the study site by consecutive sampling, while eligible normotensive controls were enrolled after matching their chronological age with those of the cases. The controls were enrolled at term just before delivery in order to avoid potential misclassification of cases and controls.

### Ethics approval and consent

We got ethical approval from the Human Research Ethics Review Committee of the University of Cape Town (ref no. 135/2018) and Walter Sisulu University (ref no. 066/2017). All participants provided informed consent before they were enrolled in the study. The study was conducted as stipulated in the Helsinki declaration.

### Data collection

After obtaining written informed consent, we used a structured questionnaire to collect the following data: participants’ age, parity, gestational age, past obstetric history of adverse pregnancy outcomes, to rule out past medical history of thyroid disease or type 1 diabetes, thyroidectomy, radioactive iodine therapy, and external radiotherapy of the head and neck. The gestational age at booking, and the highest diastolic and systolic blood pressure before enrolment, were extracted from the participants’ maternity records.

The participants’ weight was measured using a portable electronic scale and height with a portable height measuring board according to standard procedures [23]. Blood pressure at enrolment was determined by taking the average of the two measurements using an electronic sphygmomanometer at intervals ≥2 min, according to the American Heart Association guidelines [24]. Nitric oxide levels, TSH, free thyroxine (FT4), free triiodothyronine (FT3), and thyroglobulin (Tg) were assayed from venous blood collected at enrolment that was immediately centrifuged, and the serum aliquoted and stored at −20 °C until analysis. The Roche/Hitachi Cobas-c systems electrochemiluminescence immunoassay was used for determining the levels of serum TSH, FT4 and FT3. The inductively coupled plasma (ICP) Mass Spectrometry method was used to determine the urinary iodine concentration (UIC) [25] from a mid-stream urine sample collected at enrolment. The estimated daily iodine intake was determined from the UIC as described by the United States Institute of Medicine [26]. Serum nitric oxide levels were determined using the Cayman Chemical Nitrite/Nitrate Colorimetric Assay kit (Cayman Chemical Company, 1180 E. Ellsworth RD Ann Arbor, MI, USA) a two-step process where nitrate is first converted to nitrite using nitrate reductase followed by the addition of Griess Reagent 2 [N-(1-Naphthyl)ethylenediamine] that converts nitrite into a deep purple azo compound whose concentration is then determined using a colourimeter. Renal function was assessed by the determination of serum creatinine using liquid chromatography-tandem mass spectrometry. The ranges (and coefficient of variation) for various assays were TSH 0.9–11.0 IU/L (0.6), FT4 4.0–20.6 pmol/L (0.20), FT3 1.9–6.9 pmol/L (0.20), Tg 1.3–147.2 μg/L (0.78), UIC 0.0–1247.7 μg/L (1.17), Nitric oxide 0.2–12.5 μmol/L (0.52), Serum creatinine 27.0–402.0 μmol/L (0.6), and Urine iodine-creatinine ratio 0.0–2024.5 g/mol (2.3).

### Statistical analysis

Data analysis was performed using the IBM SPSS® STATISTICS software package version 22 for Windows (IBM Inc., Chicago IL, USA). We used the Shapiro–Wilk’s test to check if the data followed the normal distribution. Results are summarised as proportions (%) for categorical variables, means ± standard deviation (SD) for normally distributed, and as median (p25, p75) for skewed continuous variables, respectively. The Chi-square test was used to compare the distribution of categorical variables by status for preeclampsia. The Jonckheere-Terpstra test for trend, the Student’s t-test, Mann-Whitney U and Kruskal- Wallis tests were used for continuous variable comparisons across groups. Spearman correlation was used to determine relations between variables in the groups. Univariable and multivariable binary logistic regressions were used to investigate the correlates of preeclampsia. Thyroid function parameters categorised according to cut-off limits of normal pregnancy ranges, and potential confounders (age, parity, and BMI) were included in the regression models. The Hosmer and Lemeshow test was used to establish the goodness of fit for the logistic regression model. A *p*-value < 0.05 was significant.

## Results

### General characteristics of the participants

There was no statistical difference in BMI and gestational age at booking between the normotensive controls and cases with hypertensive diseases in pregnancy (preeclampsia and severe preeclampsia/eclampsia) (*p* > 0.05, Table 1). The respective median gestation ages (25th and 75th percentiles) for cases with preeclampsia and severe preeclampsia/eclampsia were 34 (30, 39) and 35 (32, 38) weeks compared to controls whose median gestation age at enrolment was 38 (37, 40) weeks. Participants with severe preeclampsia/eclampsia were significantly younger than their normotensive counterparts (*p* = 0.004), they also had a significantly lower number of pregnancies (gravidity) (*p* = 0.030). The highest recorded blood pressure levels during the antenatal period and the blood pressure levels at enrolment were, as expected, significantly higher among cases than controls (*p* < 0.001, Table 1).

**Table 1:** The general characteristics of normotensive pregnant controls and cases of preeclampsia and severe preeclampsia/eclampsia [median (25th and 75th percentiles)]

Variable	Normotensive (***n*** = 51)	Preeclampsia (***n*** = 51)	***P*** value*	Severe preeclampsia/ eclampsia (***n*** = 51)	***P*** value*
Age (years)	23 (17, 28)	24 (20, 29)	0.341	19 (18, 23)	0.004
BMI (kg/m^2^)	27.7 (25.5, 30.9)	29.3 (26.5, 35.3)	0.060	27.1 (23.2, 36.7)	0.250
Gravidity	1 (1, 2)	1 (1, 2)	0.941	1 (1, 1)	0.030
GA at booking (WOA)	20.1 (17.5, 23.3)	22.0 (20.0, 25.8)	0.171	22.0 (19.0, 24.0)	0.274
GA at enrolment (WOA)	38 (37, 40)	34 (30, 39)	< 0.001	35 (32, 38)	< 0.001
Highest SBP (mmHg)	121 (114, 127)	151 (145, 160)	< 0.001	162 (154, 174)	< 0.001
Highest DBP (mmHg)	74 (60, 78)	100 (90, 109)	< 0.001	102 (94, 114)	< 0.001
SBPe (mmHg)	123 (113, 130)	139 (126, 146)	< 0.001	144 (130, 151)	< 0.001
DBPe (mmHg)	77 (70, 84)	90 (81, 99)	< 0.001	92 (81, 99)	< 0.001
NO (μmol/L)	5.2 (4.0, 7.5)	4.2 (4.0, 5.1)	0.001	3.4 (1.8, 4.6)	< 0.001

*P value: Mann-Whitney test normotensive vs preeclampsia; **P value: Mann-Whitney test normotensive vs severe preeclampsia/eclampsia

*GA* gestational age, *WOA* weeks of amenorrhoea, *BMI* body mass index, *SBP* systolic blood pressure, *DBP* diastolic blood pressure, *SBPe* systolic blood pressure at enrolment, *DBPe* diastolic blood pressure at enrolment, *NO* nitric oxide, (p25, p75) 25th and 75th percentiles

Both women with preeclampsia and those with severe preeclampsia/eclampsia had significantly lower levels of nitric oxide than normotensive pregnant controls (Table 1).

### Renal and thyroid function, and iodine nutritional status of cases and controls

There was no difference in the median serum creatinine of normotensive pregnant controls and participants with uncomplicated preeclampsia (56.0 and 58.0 μmol/L respectively, *p* = 0.507) Although the median serum creatinine of women with severe preeclampsia was significantly higher than normotensive pregnant controls (68.0 μmol/L, *p* = 0.001, Table 2), it was still within the normal third-trimester range (35.0–80.0 μmol/L) with the 75th percentile reflecting mildly elevated serum creatinine in pregnancy. Participants with preeclampsia and severe preeclampsia/eclampsia had significantly low median UIC (respectively 127.7 and 98.8 μg/L) than controls (217.1 μg/L) (Table 2). However, it is only the participants with severe preeclampsia/eclampsia when compared to controls who had a significantly lower median estimated daily iodine intake (respectively 178.2 and 362.2 μg/day, *p* = 0.004), urine iodine/urine creatinine ratio (respectively 12.5 and 30.7 g/mol, *p* = 0.017), serum FT4 (12.8 and 14.2 pmol/L respectively, *p* < 0.001) and FT3 (4.0 and 4.8 pmol/L respectively, *p* < 0.001) but significantly higher median serum thyroglobulin (32.9 and 19.4 μg/L respectively, *p* < 0.001) (Table 2). There was no significant difference in the median serum TSH levels of both groups of cases and the controls (Table 2).

**Table 2:** Comparison of median (p25, p75) of thyroid function parameters TSH, FT3, FT4, Tg, UIC estimated daily iodine intake (EDII) of normotensive pregnant controls and cases of preeclampsia and severe preeclampsia/eclampsia

Variable	Normotensive (***n*** = 51)	Preeclampsia (***n*** = 51)	***P*** value*	Severe preeclampsia/ eclampsia (***n*** = 51)	***P*** value**
Ser Cr (μmol/L)	56.0 (42.0, 65.0)	58.0 (45.0, 115.8)	0.507	68.0 (53.0, 99.6)	0.001
UIC (μg/L)	217.1 (110.3, 374.5)	127.7 (75.7, 365.0)	0.046*	98.8 (39.9, 312.8)	0.005
EDII (μg/day)	362.2 (171.5, 662.8)	240.0 (128.5, 767.0)	0.144	178.2 (68.5, 508.2)	0.004
UI/UCr (g/mol)	30.7 (18.9, 88.5)	24.6 (9.9, 144.4)	0.321	12.5 (5.1, 72.9)	0.017
FT4 (pmol/L)	14.2 (13.0, 16.1)	13.7 (11.4, 16.0)	0.117	12.8 (11.5, 14.6)	0.001
FT3 (pmol//L)	4.8 (4.2, 5.0)	4.4 (4.0, 5.0)	0.087	4.0 (3.3, 4.7)	< 0.001
Tg (μg/L)	19.4 (12.5, 31.2)	21.4 (13.2, 36.3)	0.405	32.9 (18.8, 50.9)	< 0.001
TSH (mIU/L)	2.3 (1.7, 3.1)	2.3 (1.9, 3.3)	0.443	2.5 (1.6, 3.7)	0.424

*P value: Mann-Whitney U Test normotensive vs preeclampsia; **P value: Mann-Whitney U Test normotensive vs severe preeclampsia/eclampsia

*Ser Cr* serum creatinine, *UIC* urinary iodine concentration, *EDII* estimated daily iodine intake, *UI/UCr* urine iodine-creatinine ratio, *FT4 free* thyroxine, *FT3* free Triiodothyronine, *Tg* Thyroglobulin, *TSH* thyroid-stimulating hormone

We used the second and third-trimester upper serum TSH limit of 4.0 IU/L [27] and the 10th FT4 percentile of 11.3 pmol/L [28] to determine the thyroid function status of the participants. The prevalence of subclinical hypothyroidism (SCH) was 11.8, 11.8 and 15.7% for normotensive pregnant women, preeclampsia and severe preeclampsia/eclampsia participants. The respective prevalence of overt hypothyroidism for the three groups was (OH) 2.0%, 3.9.0 and 3.9% (*p* = 0.235). Hence, there was no significant difference between the groups and no participants with overt or subclinical hyperthyroidism (Table 3).

**Table 3:** Thyroid function status of normotensive, preeclamptic, severe preeclampsia/ eclamptic participants

Thyroid status	Normotensive n (%)	Preeclampsia n (%)	Severe preeclampsia/eclampsia n (%)	Chi square	***P*** value
Euthyroid	42 (82.3)	34(66.7)	31 (60.8)	8.042	0.235
SCH	6 (11.8)	6 (11.8)	8 (15.7)		
Hypothyroxinaemia	2 (3.9)	9 (17.6)	10 (19.6)		
Overt Hypothyroidism	1 (2.0)	2 (3.9)	2 (3.9)		
Total	51 (100)	51 (100)	51 (100)		

*SCH* subclinical hypothyroidism

The levels of serum FT3, FT4, urinary iodine and urine iodine/creatinine ratio showed a significant diminishing trend while serum thyroglobulin showed an increasing trend with the severity of hypertensive disease in pregnancy. Serum TSH levels showed a modest non-significant increase along the gradient of severity of hypertensive disease in pregnancy (Tables 2 and 4).

**Table 4:** Jonckheere-Terpstra test for trend of various variables along the gradient of severity of preeclampsia (normotensive, preeclampsia and severe preeclampsia/eclampsia)

Variable	Standard J-T statistic	***P*** value for trend
Highest SBP	10.19	< 0.001
Highest DBP	8.64	< 0.001
TSH	0.836	0.403
Tg	3.60	< 0.001
FT4	−3.17	0.002
FT3	−4.26	< 0.001
UIC	−2.97	0.003
Nitric oxide	−4.76	< 0.001
Serum creatinine	3.26	0.001
Urine iodine-creatinine ratio	−2.43	0.015

*SBP* systolic blood pressure, *DBP* diastolic blood pressure, *TSH* thyroid-stimulating hormone, *Tg* Thyroglobulin, *FT3* free Triiodothyronine, *UIC* urinary iodine concentration, *FT4* free thyroxine

### Correlates of preeclampsia

Participants with preeclampsia/severe preeclampsia/eclampsia were combined into a single group for comparison with normotensive pregnant controls using univariable and multivariable binary logistic regressions to establish if UIC was an independent predictor of preeclampsia-eclampsia syndrome. In order to determine the clinical relevance, we used cut-offs that are associated with preeclampsia for potential confounders, such as chronological age, gravidity, and BMI; and cut-offs associated with the normal ranges of thyroid function in pregnancy and thyroglobulin cut-off of 16.0 μg/L that reflect normal thyroid function among women with a normal pregnancy in all the three trimesters of pregnancy [2, 29]. In binary logistic regression models UIC, serum thyroxine and thyroglobulin and were significantly associated with increased odds of preeclampsia-eclampsia syndrome after adjusting for age, gravidity, BMI, TSH, FT3 (Table 5), Hosmer-Lemeshow test chi-square 14.54, *p* = 0.07).

**Table 5:** Univariable and multivariable odds ratios of urinary iodine concentration, thyroid hormones and other factors that are associated with preeclampsia-eclampsia syndrome

Variable	Univariable OR (95% CI)	***P*** value	Multivariable OR (95% CI)	***P*** value
Age < 20 yrs	1.51 (0.75–3.01)	0.248	1.10 (0.40–3.03)	0.857
Gravidity > 1	0.59 (0.30–1.17)	0.128	0.52 (0.20–1.37)	0.188
BMI > 30 Kg/m^2^	1.04 (0.52–2.12)	0.905	1.72 (0.70–4.23)	0.239
TSH > 4.0 IU/L	1.35 (0.52–3.47)	0.537	1.19 (0.41–3.47)	0.747
FT3 < 4.3 pmol/L	2.34 (1.16–4.72)	0.017	2.12 (0.98–4.63)	0.057
FT4 < 11.3 pmol/L	4.02 (1.46–11.08)	0.007	4.61 (1.48–14.42)	0.009
Tg > 16 μg/L	2.10 (1.02–4.33)	0.045	2.47 (1.02–5.99)	0.045
UIC ≤ 100 μg/L	3.37 (1.52–7.46)	0.003	2.75 (1.19–6.41)	0.018

*BMI* Body mass index, *TSH* Thyroid-stimulating hormone, *FT3* Triiodothyronine, *FT4* Thyroxine, *Tg* Thyroglobulin, *UIC* urine iodine concentration, *OR* odds ratio, *CI* confidence interval

## Discussion

This study found that women with preeclampsia had insufficient iodine intake (median urinary iodine concentration < 150.0 μg/L) while normotensive pregnant women from the same setting had adequate iodine intake in pregnancy. The degree of insufficient iodine nutrition increased while serum nitric oxide levels decreased with the severity of preeclampsia. In addition, low urinary iodine concentration, low serum thyroxine and high serum thyroglobulin were independent predictors of hypertensive disease in pregnancy in the study population. These findings correlate with previous case-control studies that reported a positive association between insufficient iodine intake and preeclampsia [12, 30, 31]. A recent systematic review found a significant difference in the mean UIC of preeclamptic and normotensive pregnant controls but no increased risk of preeclampsia among pregnant women with UIC < 150 μg/L in the included cohort studies [32]. The study was, however, limited by the very few numbers of included studies and high heterogeneity across studies. Reische et al. [33] in a recent case-control study using nationally representative data from Finland reported no difference in serum iodine, TSH and other thyroid parameters of preeclamptic and normotensive pregnant women. Differences in the findings between these studies and ours could partly be accounted for by the timing of the iodine and thyroid function tests. While the iodine and thyroid function status in our study were measured in the third trimester, the cohort studies in the systematic review [32], as well as the study by Richie et al. [33], estimated the risk of preeclampsia based only on the iodine and thyroid function status estimates in the first trimester. This approach did not consider the impact of the iodine nutritional state in the second half of pregnancy on the risk of preeclampsia. The iodine nutrition status in the first trimester may not accurately predict the iodine nutrition status in the second half of pregnancy as it depends on the daily iodine intake, renal filtration and transfer of iodine to the foetus both of which increase with gestational age [13]. It has been reported that iodine deficiency in the first trimester affects trophoblastic migration that may cause poor placentation and an ischaemic placenta, which is part of the pathophysiology of preeclampsia [34, 35]. However, an adequate iodine nutrition status in the second half of pregnancy might mitigate against the elevated oxidative stress levels arising from poor placentation and ischaemia in the first trimester [12]. Hence, optimum iodine intake in the second half of pregnancy, by increasing the serum antioxidant capacity, may help reduce the incidence and severity of preeclampsia. The risk of preeclampsia secondary to iodine deficiency may also be affected by the bio-availability of iodine which is not only dependent on the level of iodine in the diet but also on the difference in the geographical distribution of environmental factors such as nitrites, perchlorate and thiocyanates that compete with iodine at the sodium-iodine symporter in the gut and in the thyroid gland [36, 37].

Despite these apparently conflicting studies, the results of our study are in line with the findings of Abel et al. [11] who in a recent Norwegian Cohort study reported the risk of preeclampsia to increase with the degree of inadequate iodine intake. In the same study, supplementation with iodine before the onset of pregnancy reduced the risk of preeclampsia (odds ratio 0.85 (95% CI 0.74, 0.98). It seems plausible that iodine deficiency predisposes to increased risk and severity of preeclampsia at higher degrees of iodine deficiency. Our results reveal that even though participants with uncomplicated preeclampsia had lower UIC and higher Tg than normotensive controls, these were not significantly different and were within the range of mild clinical derangement. However, women with preeclampsia complicated with severe features had thyroid function parameters suggestive of moderate iodine deficiency with significantly elevated Tg that suggests longer exposure to iodine deficiency. Considering the results of Abel et al. [11], the exposure to iodine deficiency among women with severe preeclampsia in our study may have predated the current pregnancy.

Iodine is one of the exogenous scavengers of oxidative molecules produced during various metabolic processes [38, 39]. Compared to the first two trimesters of pregnancy, women in the third trimester have a higher metabolic rate and are predisposed to greater iodine loss due to increased renal filtration [40, 41]. Hence, women with low thyroid iodine content at the inception of pregnancy will be at risk of developing oxidative imbalance with resultant reduction in nitric oxide, the principal mediator of endothelial relaxation [42, 43]. This may in part explain the findings in the current study where the levels of systolic and diastolic blood pressure increased while the urinary iodine concentration and serum nitric oxide reduced along the gradient of the severity of preeclampsia.

Although spot UIC is not a reliable predictor of prolonged exposure to inadequate iodine nutrition [44], the significantly higher serum thyroglobulin levels among women with severe preeclampsia/eclampsia compared to normotensive pregnant controls in the current study seems to suggest prolonged iodine deficiency. During the acute phase of iodine deficiency, the thyroid gland undergoes autoregulation that is independent of TSH in which there is increased vascularity and iodide uptake and maintenance of physiological levels of T3 and T4 [45]. If mild-to-moderate iodine deficiency persists, apart from preferential T3 production, the thyroid gland increases the production of less iodinated thyroglobulin, which easily leaks into the bloodstream [46, 47]. This may account for the higher serum thyroglobulin observed among preeclamptic participants in the current study, who also had UIC levels suggestive of moderate iodine deficiency.

The significantly lower FT3 and FT4 against a background of low UIC among women with preeclampsia, when compared to normotensive women, suggests inadequate iodination of thyroglobulin secondary to iodine deficiency [45, 47]. However, the levels of serum FT3 and FT4 of the women with preeclampsia were still within physiological ranges, hence the mild but non-significant elevation in TSH. Previous research has revealed that high serum TSH is associated with endothelial dysfunction [48]. The proposed mechanism is the protracted stimulation of endothelial TSH receptors that is associated with diminished endothelial NO synthase activity, ultimately leading to vasoconstriction and reduced flow-mediated dilatation [49, 50].

### Strengths and limitations

Including three comparison groups of varying severity of preeclampsia in the current study and the concurrent measurement of urinary iodine concentration and serum thyroglobulin has enabled an adequate assessment of the influence of the level of iodine deficiency on the severity of preeclampsia. The study is, however, limited by our inability to measure markers of thyroid autoimmune status such as Thyroid peroxidase antibody, and serum selenium levels, another micronutrient whose deficiency is associated with thyroid dysfunction. In addition, we could not collect some clinical data such as foetal weight that would have enabled us to detect foetal growth restriction, a criterion for diagnosis of preeclampsia. Other limitations include the significant difference in gestation age at enrolment and the inability to assess the influence of iodine status on post-partum preeclampsia. Some study variables had high coefficients of variation which may have arisen from the heterogeneous nature of the 3 study groups with diverse levels of pathology.

## Conclusion

Iodine deficiency, diagnosed in the current study through low UIC, resultant hypothyroxinaemia and elevated thyroglobulin, are independent predictors of preeclampsia. Specifically, iodine deficiency that is severe enough to lead to hypothyroxinaemia and elevated thyroglobulin was associated with an increased risk of severe preeclampsia/eclampsia in the study population.

## Data Availability

The dataset generated and analysed during the current study are available in the University of Cape Town Ziva hub: Open data repository [DIO 10.25375/uct.14169587]. All data generated or analysed during this study are included in this published article.

## Abbreviations

HDP: Hypertensive disease in pregnancy
FT3: Free serum triiodothyronine
FT4: Free serum thyroxine
T3: Triiodothyronine
T4: Thyroxine
Tg: Thyroglobulin
TSH: Thyroid-stimulating hormone
UIC: Urine Iodine Concentration
